# Effect of dietary β-mannanase supplementation on growth performance, digestibility, and gene expression levels of *Cyprinus carpio* (Linnaeus) fingerlings fed a plant protein-rich diet

**DOI:** 10.3389/fvets.2022.956054

**Published:** 2022-09-02

**Authors:** Aneesa Dawood, Weibin Shi

**Affiliations:** ^1^Department of Zoology, Quaid-i-Azam University, Islamabad, Pakistan; ^2^Department of Radiology and Medical Imaging, University of Virginia, Charlottesville, VA, United States

**Keywords:** feed additive, gene expression, Non-starch polysaccharides, digestibility, mannanase, plant-protein rich diet, digestive enzymes, *Cyprinus carpio*

## Abstract

The aim of this study was to assess possible beneficial effects of dietary β-mannanase supplementation on the nutrient digestibility, growth performance, digestive and metabolic enzyme activity, and immune response of common carp (*Cyprinus carpio*) fed plant protein-rich diets. An experiment was conducted in triplicate, and a total of 225 fingerlings of common carp with an average body weight of 13.17 ± 0.12 g were stocked in 15 fiberglass tanks (15 fish/tank). Five dietary treatments (control 35% crude protein, plant-rich basal diet without supplement and four diets supplemented with β-mannanase from two sources (commercially available and locally isolated), each at two dosage levels (500 and 1,000 U/kg diet) were prepared and fed to respective groups of fish, twice a day (8:00 AM and 4:00 PM) at 4 % body weight. During the trial, changes in the level of DO and temperature ranged from 5.5 to 6.1 mg L^−1^ and 21.5 to 23.5°C, respectively. At the end of the feeding experiment, all fish in each tank were weighed and counted to determine growth parameters, while for the study of other indices, nine samples/treatment group were selected. The results of the study indicated a positive effect of both sources and dosage levels of β-mannanase supplementation on all studied indices, that is, significantly improved (*P* < 0.05), growth performance (%weight gain, specific growth rate), survival %, hematological indices (RBC, Hb, HCT, and MCHC), immunological indices (lysozyme activity, WBC, respiratory burst activity, and phagocytic activity), improved apparent digestibility of nutrients (crude protein, crude fat, and carbohydrates), and digestible energy. Furthermore, higher activity (*P* < 0.05) of the digestive enzymes (cellulase, lipase, and protease) and upregulation of MyoD gene in muscle and TNF-α gene in liver, intestine, and muscle were also observed, while the activity of serum AST (serum aspartate aminotransferase) and ALT (alanine transaminase) as compared to control group was significantly decreased (*P* < 0.05). Based on the results, β-mannanase supplementation (500 U/kg) could be recommended for obtaining better carp production when low-cost plant protein-rich diets are used.

## Introduction

Fishmeal is an excellent protein source for aquafeeds. It has a high level of crude protein (65 to 72%) with an ideal proportion of all 10 essential amino acids, omega-3 fatty acids, minerals, and certain vitamins (1). However, recent increases in aquaculture production, coupled with the high cost and limited supply of fishmeal, have resulted in an increased demand for low-cost feed alternatives that can work as well as or nearly as well as fishmeal (2). Therefore, for the sustainability of global aquaculture, appropriate alternatives to fishmeal are required.

Currently, among many sources of protein, plant-based feeds appear as easily available and cheaper alternatives to fishmeal (3, 4). At present, the global production of cereal grains stands at 2,790 million tons, legumes at 150 million tons, and fibrous materials at ~115 million tons (5). The carbohydrate content of legumes and grains is a cheap source of energy, but compared to proteins and lipids, it is poorly utilized by most fish species (6). Moreover, plant-based feed ingredients, like soybean meal, rapeseed meal, corn glutton meal, sunflower meal, root, tuber meal, and legume seeds, contain anti-nutritional factors like tannins, protease inhibitors, saponins, lectins, phytates, and non-starch polysaccharides (NSP) that can adversely affect the health and growth performance of fish (4).

Non-starch polysaccharides (NSPs) are complex, high molecular weight carbohydrates that occur naturally in plant cell walls and are one of the most important anti-nutritional agents in plant protein-rich animal feeds. NSPs increase the viscosity and resident time of digesta, affect the physiology of the gut, and alter the microbial ecosystem (3, 7). Among NSPs, mannan occurs in the form of glucomannan and galactomannan (8). Mannan is often dense and highly insoluble in water. It is present in many plant-derived feed ingredients like soybean meal, cottonseed meal, and wheat middling and interferes with the nutrient absorption in both fish and various other monogastric animals (9). Soybean meal (SBM), which is extensively used as a preferred protein source in aquafeeds, contains ~1.6 or 1.3% mannan in non-dehulled or dehulled SBM, respectively. Studies carried out on broilers, pigs, and other monogastric animals suggest that mannan reduces the digestibility and absorption of nutrients and subsequent production (3, 9). Therefore, there is an increasing interest in degradation of mannan in the plant-based diet by feed enzymes and improving the digestibility and availability of nutrients.

The dietary β-mannanase enzyme, according to the literature, can degrade mannan in plant-based diets into short-chain oligosaccharides that act as prebiotics (8, 9). Prebiotics are not digested by the host animal but metabolized by *Lactobacillus* and *Bifidobacterium* (10, 11). Such bacteria are considered beneficial for the health of the host because they reduce the risk of pathogen proliferation through the production of antibodies and antimicrobial peptides (12). Beneficial bacteria or probiotics have also been reported to enhance appetite, increase absorption of nutrients, and fortify the immune system of fish (13).

Several studies on pigs (14, 15), turkeys (16, 17), broilers (18–20), and tilapia (9) reported the importance of the supplementation of β-mannanase to enhance the digestibility of nutrients, growth rate, immune response, and overall health in these animals. However, very few studies were carried out to assess the effect of a plant protein-rich β-mannanase supplemented diet in finfish and shellfish (9, 21). In addition, mannanases from different sources may have different biophysical and biochemical properties, such as sensitivity to pepsin and pH activity profile, which can affect the *in vivo* bio efficacy of β-mannanase. There is a scarcity of comparative studies on mannanase from different origins, even though this information could be of importance for future developments of mannanase preparations and applications. In addition, a research study carried out on the comparison of mannanases from different microbial sources could provide us with evidence of a mannanase microbial source that is better. In addition, a research study carried out on the comparison of mannanases from different microbial sources could provide us with evidence of a mannanase microbial source that is better. Therefore, this study was carried out to investigate the effect of a plant protein-rich β-mannanase (obtained from two different microbial sources) supplemented diet on the performance of *C. carpio* by evaluating the growth performance, apparent digestibility of nutrients, immuno-hematological response, muscle proximate composition, the activity of digestive and metabolic enzymes, and the expression of growth and immunity-related genes (MyoD and TNF-α, respectively). Common carp was chosen for this research study because of its high food value and complicated paleotetraploidized genome. *C. carpio* is one of the most important cyprinid species. It is cultured in over 100 countries, with an annual global production of more than 4.56 million metric tons. This accounts for ~10% of worldwide freshwater aquaculture production (22).

## Materials and methods

### Fish collection and management

Healthy juvenile *C. carpio* were transferred from a local fish farm to the Fisheries and Aquaculture Research Facility, Quaid-I-Azam University, Islamabad, Pakistan. Fish were transported by adopting the live hauling technique. They were placed in plastic bags; one-third of the container was filled with water and the remaining with oxygen. After tempering, the fish were stocked in a concrete raceway system and acclimatized for 2 weeks. During this period, carps were hand-fed a 35% CP control diet (Table 1) twice a day to apparent satiation.

**Table 1:** Formulation and proximate composition of 35% crude protein basal diet (% dry matter).

**Ingredients**	**Quantity (g/100 g)**
Fishmeal	5
Soybean meal	60
Sunflower meal	10
Wheat bran	8
Rice bran	8.0
Fish Oil	1
Vitamin-mineral mixture^a^	3
DCP^b^	3.5
CMC^c^	1
Chromium oxide	0.5
**Proximate composition (%)**
Crude protein	34.36
Crude fat	12.90
NFE^d^	42.12
Chromium oxide	0.41
Crude fiber	5.32
Crude ash	5.30
Gross energy (KJ g^−1^)	21.08

a Composition of vitamin–mineral mixture (quantity/kg): vitamin B1 = 12 mg; vitamin B_6_ = 6 mg; vitamin K3 = 5 mg; vitamin B12 = 0.05 mg; vitamin B_8_ = 100 mg; vitamin B_3_ = 35 mg; vitamin B_5_ = 30 mg; folic acid, 2 mg, vitamin B_7_ = 0.06 mg; vitamin A = 25 mg; vitamin D3 = 5 mg; vitamin E = 40 mg; vitamin C = 500 mg; ethoxyquin 150 mg, wheat middling = 19,000 mg. Potassium chloride = 200 mg; potassium iodide = 60 mg; cobalt chloride = 7 mg; copper sulfate = 14 mg; iron sulfate = 400 mg; zinc sulfate = 200 mg; manganese sulfate = 80 mg; choline chloride = 100 mg; sodium selenite = 65 mg; magnesium sulfate = 3,000 mg; calcium dihydrogen phosphate = 20 g; sodium chloride = 136 mg; zeolite = 584 mg.

b DCP, dicalcium phosphate.

c CMC, carboxylmethyl cellulose.

d NFE, nitrogen-free extract.

### Preparation of diets

The commercial β-mannanase (BM_Tr_), a proprietary *Trichoderma reesei* fermentation product, having enzyme activity of 20,000 Uml^−1^ at 37°C and pH 5.5, was supplied by Youtell Biochemical Co., Ltd, Beijing, China, while locally isolated β-mannanase (BM_An_), having 78 Uml^−1^ activity at 37°C and pH 6.0, was isolated in our laboratory from a fungal strain, *Aspergillus niger* AD-01. The strain was screened from garden soil through an enrichment procedure that involved the use of media and conditions of cultivation favoring the growth of mannan-degrading species. After isolation and production of mannanase enzyme from *Aspergillus niger* AD-01, the fungal crude enzyme was purified by two-step purification, that is, ammonium sulfate precipitation and gel-filtration chromatography. The detailed procedure of isolation and purification has been reported earlier (23). Ingredients for feed were obtained from a feed mill (Oryza Organics Pvt. Ltd., Lahore, Pakistan).

A plant protein-rich basal diet containing soybean meal as a major ingredient was formulated. Its proximate composition is shown in Table 1. Five tested diets were prepared where β-mannanase was added to the basal diet at a dose of 0 units kg^−1^ diet (control group), and 500 and 1,000 units kg^−1^ diet BM_Tr_ (BM_Tr500_ and BM_Tr1,000_, respectively) and BM_An_ (BM_An500_ and BM_An1,000_), respectively. Briefly, all dry ingredients except vitamin and mineral premix were finely ground with the help of an electric grinder (GCG289, Geepas electronics, Dubai). Afterward, to obtain 35% CP, different feed ingredients were mixed along with vitamin–mineral premix in a fixed ratio (Table 1) by using a locally made horizontal livestock feed mixer (24). Subsequently, oil and water were added to the ingredients and made into a dough. The dough was passed through a meat grinder, and the resultant noodles were cut into small pellets, which were air-dried at room temperature (25°C). For β-mannanase supplementation, the required amount of enzyme (both commercial and extracted) was dissolved in distilled water and sprayed on experimental diets, while the control diet was sprayed with just the distilled water of the same amount. Each group of feed was dried once again at room temperature (25°C). The pellets were transferred to a Ziploc bag and stored in a refrigerator. Fortnightly, a new batch of feed was prepared.

### Experimental design

A bi-factorial feeding trial (3 β-mannanase dosage levels × 2 enzyme sources) in a replicate of three was designed. Fish were weighed, and 225 carps (*C. carpio), with a* mean body weight of 13.17 ± 0.12 g, were selected randomly and equally distributed into fifteen 250 L circular fiberglass tanks (15 fish per tank with three replicates per treatment) at a stocking density of 1.5 gL^−1^. The fish were acclimatized to laboratory conditions for 3 days. Afterward, tanks were randomly divided into five groups, and each group was provided with their respective diet, i.e., the control group was fed β-mannanase free basal diet, the BM_Tr500_ and BM_Tr1,000_ groups of fish received a basal diet supplemented with 500 and 1000 units of BM_Tr_ kg^−1^ diet, respectively, while the BM_An500_ and BM_An1,000_ groups of fish were provided with a basal diet enriched with 500 and 1,000 units of BM_An_kg^−1^ diet, respectively. Fish were fed their respective diets twice a day (8:00 am and 4:00 pm) for 90 days. Initially, they were fed at 4% body weight per day, and fortnightly, fish from each group were weighed individually by using an electronic balance (Shimadzu BL2200H, Japan), and the feeding rate was adjusted accordingly. During the trial, the temperature (°C), dissolved oxygen (DO) level (mg L^−1^), and pH of water were checked daily by using the Hanna water testing instrument (HI-9828; Inc. Woonsocket, USA). However, total ammonia was checked weekly using an ammonia test kit (Hanna HI-3824, Romania). During the experimental period, the DO level ranged from 5.5 to 6.1 mgL^−1^ while the temperature increased from 21.5 to 23.5°C. However, no noticeable variations in other parameters were observed, that is, pH (7.2 ± 0.52) and total ammonia (<0.5 ppm). To maintain optimum water quality and for the calculation of FCR (feed conversion ratio), daily uneaten feed and feces of each tank were collected after 2 and 6 h of feeding, respectively, with a partial change of water. Since all tanks were adjacent to one another and had a similar source of water, the variation in water quality parameters was negligible. After 90 days, the fish were fasted for 24 h prior to sampling.

### Nutrient digestibility

For estimating the apparent digestibility coefficient of nutrients, the inert indicator method was used. Briefly, after 60 days of experiment, fish were fed with diets having 0.5% chromic oxide (Cr_2_O_3_). For apparent digestibility coefficient (ADC) of nutrients, daily uneaten feed and feces of each tank were collected through siphoning after 2 and 6 h of feeding, respectively, with partial change of water. The collected fecal samples of each group were filtered, dried at 60°C in a preheated oven, and stored at −20°C. Fecal collection continued for 30 days. For analysis, fecal samples of each group were pooled (15-day samples/pool, thus six samples/experimental group), dried in oven, and homogenized by using a pestle and motor. Chemical analysis of feed and face samples was performed by adopting AOAC (2000) procedure. Dry matter (DM) was determined by oven drying at 105°C for 16 h, crude protein (CP) by micro-Kjeldahl analysis, and gross energy by oxygen bomb calorimeter. Crude fat was determined following Bligh and Dyer (24) petroleum ether extraction method by 10454 soxtec system HTz. Chromium oxide content of diet and feces samples were analyzed by the method described by Divakaran et al. (25), by UV-VIS 2001 spectrophotometer.

For *C. carpio*, apparent digestibility coefficients of crude protein, crude fat, and carbohydrates of the experimental diets were determined by using the following equation:


$$\begin{array}{l} \text{ADC}\left(\text{\%}\right)=100-\left[\frac{\text{\% Cr in feed}}{\text{Cr in feces}}\times \frac{\text{\% nutrient in feces}}{\text{\%nutrient in feed}}\right]\times 100\% \end{array}$$

### Survival rate and growth performances

On the day of sampling, fish from each tank were weighed collectively and counted to determine the mean body weight and survival rate (100 × Final number of fish/initial number of fish). Based on observed initial and final weight of fish, the growth performance parameters like specific growth rate (SGR (%) = (ln final weight −ln initial weight) × 100/Total experimental days), percent weight gain (WG % = final body weight- initial body weight/ initial body weight × 100), and feed conversion ratio (FCR = dry weight of feed consumed/wet weight gain) of each group were also calculated.

### Sample collection

Thirty six fish from each group (12 fish from each replicate) were randomly captured and anesthetized with buffered tricaine methanesulfonate (MS-222, 0.10 gL^−1^). The blood of 18 fish (six fish per replicate) was drawn from the caudal vein by using a 3 mL sterile syringe. To collect enough serum, blood from two fish was pooled in the same tube (three samples/tank or nine samples/treatment group) and allowed to clot at room temperature. Subsequently, the clotted blood was centrifuged (Kokusan Ogawa Seiki Co., LTD, Tokyo, Japan) at 3,000 rpm for 5 min, and the blood serum was collected in a new tube and saved at 4°C until further analysis. For hematological analysis, the blood of the other 18 fish (6 fish/tank) was collected from the caudal vein with a 3 mL syringe previously rinsed with EDTA (2.7% solution) and collected in EDTA tubes (Liuyang Sanli Medical Technology Development Co., LTD). Again, the blood of two fish was pooled (three blood samples from each replicate or nine samples /treatment group).

After being bled, six fish per tank were dissected at a low temperature by following a standard aseptic procedure, and their intestinal tracts were removed carefully. The guts of six fish per tank, a pool of two (three samples/tank or nine samples/treatment group), were frozen quickly in liquid nitrogen and saved at −80°C for later determination of intestinal enzyme activities. Similarly, the muscles of each group of fish after blood sampling were saved for the study of proximate composition. Moreover, for investigation of gene expression, the liver, muscle, and intestinal tissues of three fish per tank were preserved in RNA later and stored at −80°C.

### Chemical composition of muscle, feed, and feces

For chemical analysis of fish muscle, experimental diets and fecal samples were sent to the accredited laboratory of the Poultry Research Institute (PRI), where they were analyzed by adopting a standard protocol (25).

Briefly, for the determination of ash content, incineration in a muffle furnace was carried at 600°C for 12 h. The micro-Kjeldahl apparatus and the petroleum ether extraction method by Soxhlet apparatus (26) were used to estimate crude protein (N × 6.25) and crude fat, respectively. Oxygen bomb calorimeter was used for the determination of gross energy. Chromic oxide content in feces and experimental diets was determined after oxidation with perchloric reagent by using the acid digestion method as described by Divakaran et al. (27) through the UV-VIS 2001 spectrophotometer.

### Digestive enzyme activity

The collected intestines were homogenized in 10 mL of chilled phosphate-buffered saline (pH, 7.5) using a handheld homogenizer. Subsequently, the homogenate was centrifuged at 3000 × g for 10 min at a low temperature (4°C), and the supernatant was collected in a new tube and saved at −80°C for subsequent digestive enzyme analysis.

#### Amylase activity

For amylase assay, the DNS (3,5 dinitrosalicylic acid) method based on the determination of reducing sugars at 560 nm, using maltose as standard, was used (28). Briefly, 0.5 mL of enzyme solution was mixed with an equal amount of starch solution (1%) and was left to incubate at room temperature for 3–5 min. Afterward, 1 mL of DNS reagent (prepared by mixing DNS = 1 g, 2N sodium hydroxide = 20 mL, and sodium potassium tartrate = 30 g and diluted to 100 mL with distilled water) was added and left to incubate in a preheated water bath for at least 5 min. The mixture was cooled and mixed with 10 mL of reagent graded water. The UV-Visible spectrophotometer (Agilent, 8453, USA) was set at λ 450 nm, and the absorption of the resultant solution was noted for estimating amylase activity. One amylase unit was defined as the amount of enzyme in 1.0 mL of solution that released 1 μg of reducing sugar per min.

#### Cellulase activity

The production of reducing sugars as a result of cellulolytic activity was measured by adopting the DNS method. Briefly, 1 mL each of enzyme solution and CMC solution (1%) were taken in a 10 mL glass tube and mixed with 1 mL of 0.1 M citrate buffer. The resultant solution was left to incubate at 50°C for half an hour. Afterward, 3.0 mL of DNS reagent was added and incubated at the boiling point for 15 min. Subsequently, 1 mL of 40% sodium potassium tartarate was added to the solution, and the test tube was cooled at room temperature. The UV-Visible spectrophotometer (Agilent, 8453, USA) was set at λ 540 nm, and reducing sugar, i.e., glucose, was measured for estimating the cellulolytic activity. One cellulase unit was defined as the quantity of enzyme per mL solution that released 1 mg of glucose per min.

#### Protease activity

The protease activity was analyzed by using casein as a substrate and L-tyrosine for the preparation of a standard curve (29). Briefly, 100 μL of enzyme extract was mixed with 0.65% of casein solution (5 ml) and 110 mM of trichloroacetic acid (5 mL). The resultant solution was left to incubate at 37°C for half an hour and then cooled at room temperature. Afterward, the solution was filtered through Whatman filter paper. Subsequently, 2.0 mL of filtrate was taken in a separate clean 10 mL glass test tube and mixed with 5 mL of sodium carbonate solution (500 mM) and 1 mL of Folin–Ciocalteu reagent (0.5 mM) and heated for 30 min at 37°C and then cooled at room temperature. The UV-Visible spectrophotometer (Agilent, 8453, USA) was set at λ 660 nm, and the absorption of the resultant solution was noted for calculating protease activity. One unit of enzyme activity was defined as the quantity of enzyme required to liberate 1 μg of tyrosine per mL filtrate under standard assay conditions.

### Hematological indices

Hematological indices like WBCs (white blood cells), RBCs (red blood cells), MCV (Mean corpuscular volume), MCH (mean corpuscular hemoglobin), Hct (hematocrit), MCHC (mean corpuscular hemoglobin concentration), and Hb (hemoglobin) were determined by using a pre-calibrated Hematology Analyzer (DxH 500, Beckman Coulter).

### Immunological and blood biochemical indices

Total serum proteins, immunoglobulin (IgM), aspartate aminotransferase (AST), and lysozyme activity were measured by adopting the protocols used by Ullah et al. (30), while the protocol reported by Anderson and Zeeman (31) was followed for the assessment of respiratory burst and phagocytic activity from fresh heparinized blood. Cholesterol (CHO) and triglyceride (TG) assays were performed by adopting standard procedures as reported by (32).

#### Total serum protein and IgM level

Total serum protein was analyzed by following Lowry's method (33), using bovine serum albumin (BSA) as a standard for the preparation of the calibration curve and the determination of protein concentrations. For the determination of serum IgM levels, Anderson and Siwicki (34) procedure with some modification was adopted. After mixing 0.1 mL of serum and 12% polyethylene glycol, the solution was incubated at room temperature for 2 h with constant shaking (ISS Innova 43 shaking incubator). Afterward, the solution was centrifuged at 7,000 rpm for 10 min, and the supernatant was separated. The protein concentration in the supernatant was determined by adopting Lowry's method, and the IgM level was calculated by subtracting the IgM value from total serum protein.

#### Lysozyme activity

To determine the lysozyme activity, Anderson and Siwicki (34) method reported by Ullah et al. (30) was followed. Briefly, serum (100 μL) was taken with the help of a micropipette in a fresh test tube and mixed with 900 μl of 750 μg mL^−1^ *Micrococcus lysodeikticus* (Sigma, USA) suspended in saline phosphate buffer (pH 6.2) solution. Bacteria were thoroughly mixed, and the rate of absorbance change was observed by using a spectrophotometer set at 540 nm. The reading was noted after 1 min interval for 10 min. Lysozyme activity was measured by using lysozyme from hen egg white (Sigma-Aldrich) as a standard.

#### Phagocytic activity

For the determination of phagocytic activity, heparinized blood (100 μL) was mixed with killed *S. aureus (1* × 107 cells) suspended in phosphate-buffered saline (pH = 7.2). The mixture was left to incubate at room temperature for half an hour. Afterward, a smear was prepared by transferring the mixture (5 μL) to a glass slide. The smear was air-dried, fixed in ethanol (95%) for 5 min, air-dried again, and then dipped in Giemsa stain. From each smear, a total of 100 phagocytic cells were read under the microscope, and the number of phagocytic cells and phagocytosed bacteria was counted. Phagocytic activity (PA) and phagocytic index (PI) were measured by the following formulas:

PA=No. of phagocytic cells with ingested bacteria/No. Of phagocytes x100

PI=No. of engulfed bacteria/phagocytic cells.

#### Respiratory burst activity

Briefly, heparinized blood (100 μL) was placed in a microplate and mixed with 100 μL of 0.2% nitro blue tetrazolium (NBT) dye. The mixture was left to incubate at room temperature for half an hour, and then, 0.05 ml of this mixture was transferred to a glass tube containing 1ml of N-N di-ethyl methyl formamide solution and centrifuged for 5 min at 3000 rpm. The supernatant's absorbance was read at 540 nm.

### Total RNA extraction and cDNA synthesis

From each sample, the total RNA was extracted using buffer RLT (Qiagen, Mississauga, Canada) and 0.12 M β-mercaptoethanol (Sigma). The RNA was extracted using RNeasy mini kit (Qiagen, Mississauga, Canada) and quantified at 260 nm. All OD_260_/OD_280_ were between 1.8 and 2.0. The cDNA was synthesized via Revert Aid First-strand cDNA synthesis kit (Thermo Fisher Scientific, Lithuania) by following the manufacturer's protocol. RNA (1 μg) along with random hexamer primers was incubated for 5 min at 70°C followed by cooling for 10 min at room temperature; thus, the primers were annealed appropriately to RNA. Following that, RT-buffer, dNTPs, RNAs inhibitor, and RT enzyme were added to the mixture, which was then incubated for 5 min at 25°C, 60 min at 42°C, and 3 min at 95°C in a thermal cycler (Master cycler, Gradient Eppendorf, USA). The resultant sample was stored at −20°C until further analysis.

#### qPCR conditions for analysis of gene expression

The housekeeping gene, β-actin, was used to examine the relative expression of the selected genes, namely MyoD in muscle and TNF-α gene in the intestine, liver, and muscle tissue of each group of fish. The sequence of primers for the reference gene and selected genes is shown in Table 2. The amplification of cDNA was performed on a LightCycler^®^ 480 instrument by using the SYBR^®^ Premix ExTaq^TM^ kit (Takara Bio, Japan). The 20 μL mixture was prepared by mixing 1.0 μL of the forward and reverse primers (10 mM), 10 μL of SYBR Premix Ex Taq™, and 1.0 μL of cDNA with 8 μL of ultra-pure water. The thermal cycling conditions involved initial denaturation at 95°C for 10 min followed by 40 cycles at the same temperature, that is, 95°C for 15s, then annealing at 56°C for 30s, and final extension at 72°C for 30s.

**Table 2:** Primers used for the expression of MyoD in muscle and TNF-α in muscle, liver, and intestine of *C. carpio*.

**Gene**		**Primer sequence (5^′^ → 3^′^)**	**Accession number**	**References**
TNF-α	F	GGTGATGGTGTCGAGGAGGAA	XM_019088899.1	(35)
	R	TGGAAAGACACCTGGCTGTA	XM_019088899.1	
Myo-D	F	TGCCTACTGTGGGCATGCAA	LN594833.1	(36)
	R	ACTCACTTCTGCTGATCTGC	LN594833.1	
β-actin	F	AGAAGGACCACTTGCACTCA	KX622693.1	(37)
	R	GATGCCAAATACTGCTCAATGT	KX622693.1	

For each sample, qPCR was run two times with three replicates. The efficiency and validity of qPCR primers were determined by Equation, %E = 10(– 1/Slope) – 1 × 100 (38). For relative gene expression, ΔΔCt method based on the expression of target gene relative to the expression of housekeeping gene (reference) was adopted by using IQ5 software (Bio-Rad).

### Statistical analysis

All data are presented as means ± standard deviation (SD). SPSS software (SPSS 18.0. Chicago, IL) was used to carry out all statistical procedures. Analysis of the data was done by two-way ANOVA followed by Fisher's *post-hoc* LSD test to determine significant differences among experimental groups. A two-tailed *t*-test was used to compare the results of both enzymes at a similar dosage levels. The value *P* < 0.05 was used as the criterion for statistical significance.

## Results

### Apparent digestibility of nutrients and digestible energy

The addition of an exogenous β-mannanase enzyme showed a significant effect on the apparent nutrient digestibility coefficients (ADC %) and digestible energy (Table 3). After a 90-day experimental period, all groups of fish fed β-mannanase supplemented diet (BM_Tr500_, BM_Tr1,000_, BM_An500_, and BM_An1,000_) showed statistically similar (*P* > 0.05) but significantly higher (*P* < 0.05) apparent nutrient digestibility coefficients of crude protein, crude fat, and carbohydrates as compared to the control group of fish. Two-way ANOVA indicated a significant effect of the dosage level of β-mannanase (*P* < 0.05) for all studied parameters, while a non-significant effect of enzyme source (*P* > 0.05) on the digestibility of crude protein, carbohydrates, and digestible energy.

**Table 3:** Effect of β-mannanase supplemented diets on apparent digestibility of major nutrients and digestible energy of common carp.

**Treatment groups**	**Crude protein**	**Crude fats**	**Carbohydrates**	**Energy**	**Digestible energy (KJ g^−1^)**
Control	70.76^c^ ± 1.8	73.6^c^ ± 2.1	64.01^c^ ± 2.1	68.16^c^ ± 1.3	14.08^c^ ± 0.5
BM_Tr500_	73.95^a^ ± 1.0	77.9^ab^ ± 2.1	67.58^ab^ ± 1.1	72.98^ab^ ± 1.6	15.66^a^ ± 0.68
BM_Tr1,000_	72.98^ab^ ± 1.0	76.78^ab^ ± 2.1	66.1^b^ ± 2.0	71.07^b^ ± 1.8	14.83^b^ ± 0.71
BM_An500_	71.8^b^ ± 1.8	76.1^b^ ± 2.1	67.36^ab^ ± 1.6	71.98^ab^ ± 1.4	15.2^ab^ ± 0.59
BM_An1,000_	73.50^ab^ ± 1.5	78.54^a^ ± 2.1	68.45^a^ ± 1.17	73.06^a^ ± 1.6	15.31^ab^ ± 0.64
**Two-way ANOVA** ***P*****-values**					
Enzyme source	0.345	0.972	0.200	0.416	0.876
Dosage level	0.031	0.001	0.001	0.001	0.001
Interaction	0.118	0.052	0.119	0.357	0.265

a, b, c Values are mean ± SD for three replicate groups of fish (n = 6) where the values within a column without a common superscript differ (p < 0.05). BM_Tr500_ and BM_Tr1,000_ designate 500 and 1,000 units/kg of commercial β-mannanase (BM_Tr_), while BM_An500_ and BM_An1,000_ designate 500 and 1,000 units/kg of extracted β-mannanase enzyme (BM_An_), respectively. Control group contains 0 units/kg of β-mannanase.

### Growth performance

The initial body weights of carp in the control group and experimental groups BM_Tr500_, BM_Tr1,000_, BM_An500_, and BM_An1,000_ were considerably similar (*P* > 0.05). However, at the end of the feeding trial, fish in groups BM_Tr500_, BM_Tr1,000_, BM_An500_, and BM_An1,000_ gained statistically similar (*P* > 0.05) but significantly more weight (*P* < 0.05) than fish in control group. Similarly, the specific growth rate (%) was also significantly higher in groups of fish reared on β-mannanase supplementation than in control group (Table 4). FCR value of all β-mannanase supplemented groups was also similar and lower than the control group.

**Table 4:** Effect of β-mannanase supplemented diets on growth performance of *C. carpio*.

**Treatment groups**	**Initial body weight (g)**	**Final body weight (g)**	**%Weight gain**	**Specific growth rate (%/day)**	**Net weight gain**	**FCR**	**Survival (%)**
Control	13.22 ± 0.28	40.7 ± 1.00^c^	207.87 ± 5.36^b^	1.25 ± 0.03^c^	26.85 ± 1.12^c^	2.35 ± 03^a^	96.3 ± 2.08^b^
BM_Tr500_	13.19 ± 0.36	46.62 ± 1.9^b^	253.49 ± 14.72^a^	1.39 ± 0.04^b^	33.43 ± 1.88^b^	1.9 ± 0.19^b^	100 ± 0.0^a^
BM_Tr1,000_	13.62 ± 0.15	49.08 ± 2.6^a^	260.16 ± 15.97^a^	1.42 ± 0.04^a^	35.45 ± 2.50^b^	1.88 ± 0.11^b^	99.1 ± 1.16^a^
BM_An500_	13.35 ± 0.28	51.44 ± 2.9^a^	285.68 ± 29.86^a^	1.49 ± 0.08^a^	38.09 ± 3.24^a^	1.86 ± 0.11^b^	99.2 ± 1.5^a^
BM_An1,000_	13.05 ± 0.20	48.1 ± 1.57^a^	268.53 ± 10.53^a^	1.44 ± 0.02^a^	35.05 ± 1.47^b^	1.83 ± 0.12^b^	98.6 ± 1.65^a^
**Two-way ANOVA** ***P*****-values**							
Source		0.205	0.104	0.111	0.167	0.737	0.408
Dosage		0.001	0.649	0.001	0.001	0.001	0.004
Source × Dosage		0.062	0.313	0.223	0.097	0.969	0.817

a, b, c Values are mean ± SD for three replicate groups of fish (n = 3) where the values within a column without a common superscript differ (p < 0.05). BM_Tr500_ and BM_Tr1,000_ designate 500 and 1,000 units/kg of commercial β-mannanase (BM_Tr_), while BM_An500_ and BM_An1,000_ designate 500 and 1,000 units/kg of extracted β-mannanase enzyme (BM_An_), respectively. Control group contains 0 units/kg of β-mannanase.

Two-way ANOVA of all studied growth-related parameters, FCR, and survival indicated a significant effect (*P* < 0.05) for dosage levels of mannanase except for % weight gain, a non-significant difference (*P* > 0.05) for enzyme source, and the interaction between two variables (enzyme dosage × enzyme sources). However, all pairwise comparisons indicated statistically similar positive effects of both enzymes at both supplementation levels.

### Muscle proximate composition

β-mannanase supplemented diet showed a beneficial effect on the muscle composition of common carp (Table 5). We observed a significant increase in the protein and fat contents and a decrease in moisture (%) in the β-mannanase supplemented groups compared to a control group of fish. However, no significant difference was found in the ash content of the control and treatment groups. Again, two-way ANOVA showed a significant difference (*P* < 0.05) among dosage levels, a non-significant difference (*P* > 0.05) among sources of β-mannanase, and an interaction between dosage levels and enzyme source (enzyme dosage × enzyme sources). However, all pairwise comparisons indicated statistically similar values of all proximate composition indices at both supplementation levels of both enzymes.

**Table 5:** Effect of β-mannanase supplemented diet on the proximate composition of muscle of *C. carpio*.

**Treatment groups**	**Moisture (%)**	**Protein content (%)**	**Fat contents (%)**	**Ash (%)**
Control	77.18 ± 0.87^a^	13.70 ± 0.74^b^	4.07 ± 1.12^b^	2.76 ± 0.25
BM_Tr500_	72.08 ± 1.79^b^	16.24 ± 0.91^a^	5.95 ± 0.54^a^	2.43 ± 0.46
BM_Tr1,000_	73.15 ± 1.45^b^	16.95 ± 0.73^a^	6.85 ± 0.82^a^	2.32 ± 0.50
BM_An500_	72.81 ± 1.35^b^	15.98 ± 1.49^a^	6.10 ± 1.27^a^	2.36 ± 0.70
BM_An1,000_	73.70 ± 1.41^b^	16.55 ± 1.2^a^	6.07 ± 0.80^a^	2.57 ± 0.64
**Two-way ANOVA** ***P*****-values**				
Source	0.245	0.470	0.374	0.677
Dosage	0.0001	0.0001	0.0001	0.087
Source × Dosage	0.695	0.862	0.242	0.619

a, b, c Values are means ± SD for three replicate groups of fish (n = 9) where the values within a column without a common superscript differ (P < 0.05). BM_Tr500_ and BM_Tr1,000_ designate 500 and 1,000 units/kg of commercial β-mannanase (BM_Tr_), while BM_An500_ and BM_An1,000_ designate 500 and 1,000 units/kg of extracted β-mannanase enzyme (BM_An_), respectively. Control group contains 0 units/kg of β-mannanase.

### Digestive enzyme activity

The activity of digestive enzymes is an indication of digestion and absorption of nutrients in the gastrointestinal tract. Here, supplementation of β-mannanase enhanced the digestive enzyme activity of common carp (Table 6). After a 90-day experimental period, all groups of fish fed β-mannanase supplemented diet (BM_Tr500_, BM_Tr1,000_, BM_An500_, and BM_An1,000_) showed statistically similar (*P* > 0.05) but significantly higher (*P* < 0.05) amylase activity compared to the control group of fish. In comparison with amylase, the activities of protease and cellulase increased with an increase in the dosage level of β-mannanase. Again, two-way ANOVA indicated an insignificant effect (*P* > 0.05) of enzyme source and interaction between dosage levels and enzyme source (enzyme dosage × enzyme sources) on intestinal enzyme activities.

**Table 6:** Effect of β-mannanase on digestive enzyme activity in *C. carpio*.

**Treatment Groups**	**Cellulase (U mg−1)**	**Protease (U mg−1)**	**Amylase (U mg−1)**
Control	0.426 ± 0.16^c^	0.34 ± 0.09^c^	0.47 ± 0.17^b^
BM_Tr500_	1.63 ± 0.24^b^	1.15 ± 0.13^b^	1.37 ± 0.27^a^
BM_Tr1,000_	2.23 ± 0.20^a^	1.29 ± 0.14^a^	1.45 ± 0.23^a^
BM_An500_	1.83 ± 0.16^b^	1.22 ± 0.11^b^	1.32 ± 0.21^a^
BM_An1,000_	2.33 ± 0.27^a^	1.43 ± 0.20^a^	1.53 ± 0.21^a^
**Two-way ANOVA** ***P*****-values**			
Source	0.230	0.114	0.905
Dosage	0.0001	0.0001	0.0001
Source × Dosage	0.302	0.443	0.924

a, b, c Values are mean ± SD for three replicate groups of fish (n = 9) where the values within a column without a common superscript differ (P < 0.05). BM_Tr500_ and BM_Tr1,000_ designate 500 and 1,000 units/kg of commercial β-mannanase (BM_Tr_), while BM_An500_ and BM_An1,000_ designate 500 and 1,000 units/kg of extracted β-mannanase enzyme (BM_An_), respectively. Control group contains 0 units/kg of β-mannanase.

### Hematological indices of carp

β-mannanase also showed a significant effect on different hematological indices of carp. The WBC, RBC, Hb, HCT (%), and MCHC levels were significantly higher (*P* < 0.05) in the β-mannanase supplemented groups than in the control group (Table 7). However, MCH and MCV showed a significantly decreased level (*P* < 0.05) in β-mannanase fed groups compared to a control group. Two-way ANOVA indicated an insignificant effect (*P* > 0.05) of enzyme source and interaction between dosage levels and enzyme source (enzyme dosage × enzyme sources) on all hematological indices of common carp. However, all pairwise comparisons indicated an almost similar positive effect of both enzymes on all hematological indices of common carp.

**Table 7:** Effect of β-mannanase on hematological indices of *C. carpio*.

**Treatment Groups**	**RBC (10^6^ μL)**	**WBC (10^3^ μL)**	**Hb (g/100 mL)**	**HCT (%)**	**MCV (10^−15^ L)**	**MCH (pg)**	**MCHC (g/100 mL)**
Control	1.56 ± 0.12^c^	142.2 ± 1.21^c^	5.14 ± 0.12^c^	19.3 ± 1.42^c^	201.66 ± 7.09^a^	33.74 ± 1.98^a^	14.72 ± 0.85^c^
BM_Tr500_	1.94 ± 0.06^b^	184.06 ± 3.8^a^	8.07 ± 0.22^a^	25.59 ± 2.58^b^	145.32 ± 9.51^c^	26.18 ± 1.85^bc^	18.79 ± 1.96^b^
BM_Tr1,000_	2.08 ± 0.13^a^	181.9 ± 2.95^a^	7.71 ± 0.33^b^	27.02 ± 2.3^b^	176.15 ± 4.47^b^	25.7 ± 2.25^c^	21.26 ± 1.4^a^
BM_An500_	1.92 ± 0.10^b^	178.2 ± 4.45^b^	7.65 ± 0.24^b^	29.59 ± 2.15^a^	174.6 ± 10.01^b^	25.93 ± 2.65^c^	18.46 ± 0.58^b^
BM_An1,000_	1.88 ± 0.10^b^	185.1 ± 4.6^a^	8.23 ± 0.66^a^	24.94 ± 2.77^b^	180.12 ± 6.44^b^	27.36 ± 2.85^b^	19.86 ± 1.15^b^
**Two-way ANOVA** ***P*****-values**							
Source	0.270	0.265	0.844	0.239	0.006	0.413	0.104
Dosage	0.001	0.001	0.001	0.001	0.0244	0.001	0.001
Source × Dosage	0.396	0.001	0.094	0.001	0.0042	0.336	0.238

a, b, c Values are means ± SD from triplicate groups of fish (n = 9) where the values within a column without a common superscript differ (p < 0.05). BM_Tr500_ and BM_Tr1,000_ designate 500 and 1,000 units/kg of commercial β-mannanase (BM_Tr_), while BM_An500_ and BM_An1,000_ designate 500 and 1,000 units/kg of extracted β-mannanase enzyme (BM_An_), respectively. Control group contains 0 units/kg of β-mannanase.

### Immunological indices

Immunological indices like lysozyme activity, respiratory burst activity, immunoglobulin, and phagocytic activity also showed a significant increase in response to β-mannanase supplemented diets. Except for phagocytic activity and phagocytic index, all studied immunological indices showed similar levels in response to both sources of β-mannanase and at both dosage levels. However, phagocytic activity and phagocytic index showed a dose-dependent increasing trend (*P* < 0.05). Similarly, a significant interaction (*P* < 0.05) between source and dosage effect was also found only on phagocytic activity (Table 8).

**Table 8:** Effect of β-mannanase supplementation on immunological indices of *C. carpio*.

**Treatment groups**	**Globulin (mg/mL)**	**Lysozyme activity (μg/mL)**	**Respiratory burst activity (OD at 540 nm)**	**Phagocytic activity (%)**	**Phagocytic index**
Control	17.82 ± 0.46^b^	1.93 ± 0.25^b^	0.28 ± 0.04^b^	23.45 ± 1.0^d^	1.38 ± 0.25^c^
BM_Tr500_	26.6 ± 2.57^a^	3.29 ± 0.20^a^	0.72 ± 0.03^a^	57.82 ± 1.93^c^	2.19 ± 0.187^b^
BM_Tr1,000_	25.3 ± 2.17^a^	3.22 ± 0.37^a^	0.81 ± 0.03^a^	64.53 ± 1.36^a^	2.45 ± 0.32^a^
BM_An500_	24.72 ± 2.70^a^	3.13 ± 0.33^a^	0.78 ± 0.07^a^	61.87 ± 1.33^b^	2.28 ± 0.414^b^
BM_An1,000_	25.53 ± 2.41^a^	3.63 ± 0.27^a^	0.74 ± 0.08^a^	63.71 ± 0.97^a^	2.35 ± 0.37^a^
**Two-way ANOVA** ***p*****-values**					
Source	0.307	0.103	0.972	0.006	0.934
Dosage	0.001	0.001	0.001	0.001	0.001
Source × Dosage	0.373	0.410	0.021	0.001	0.645

a, b, c Values are means ± SD for three replicate groups of fish (n = 9) where the values within a column without a common superscript differ (p < 0.05). BM_Tr500_ and BM_Tr1,000_ designate 500 and 1,000 units/kg of commercial β-mannanase (BM_Tr_), while BM_An500_ and BM_An1,000_ designate 500 and 1,000 units/kg of extracted β-mannanase enzyme (BM_An_), respectively. Control group contains 0 units/kg of β-mannanase.

### Biochemical indices and cholesterol level

β-mannanase supplemented diet also showed a significant effect on blood biochemical indices (Table 9). All groups of fish (BM_Tr500_, BM_Tr1,000_, BM_An500_, and BM_An1,000_) fed β-mannanase enriched diet showed a significant (*P* < 0.05) dose-dependent decrease in serum AST and ALT activity while increase in serum protein, cholesterol, and TG levels compared to the control group.

**Table 9:** Effect of β-mannanase on blood biochemical indices of *C. carpio*.

**Treatment groups**	**Total serum protein (g/100 mL)**	**ALT (UL^−1^)**	**AST (UL^−1^)**	**CHO (mg/100 mL)**	**TG (mg/100 mL)**
Control	2.91 ± 0.24^b^	25.73 ± 0.88^a^	63.26 ± 1.88^a^	10.9 ± 0.83^c^	16.02 ± 1.2^c^
BM_Tr500_	3.71 ± 0.21^a^	19.04 ± 0.65^b^	48.21 ± 0.92^b^	11.27 ± 1.02^b^	16.9 ± 0.98^b^
BM_Tr1,000_	3.90 ± 0.22^a^	17.82 ± 1.05^c^	46.29 ± 1.11^c^	12.75 ± 0.89^a^	17.8 ± 1.06^a^
BM_An500_	3.76 ± 0.188^a^	18.13 ± 1.0^bc^	48.66 ± 0.90^b^	11.53 ± 0.94^b^	17.4 ± 0.74^ab^
BM_An1,000_	3.62 ± 0.36^a^	18.86 ± 0.85^b^	47.60 ± 1.96^bc^	13.04 ± 0.90^a^	18.2 ± 0.95^a^
**Two-way ANOVA** ***P*****-value**					
Source	0.304	0.877	0.175	0.574	0.297
Dosage	0.001	0.001	0.001	0.001	0.001
Source × dosage	0.230	0.02	0.446	0.922	0.750

a, b, c Values are means ± SD for three replicate groups of fish (n = 9) where the values within a column without a common superscript differ (p < 0.05). BM_Tr500_ and BM_Tr1,000_ designate 500 and 1,000 units/kg of commercial β-mannanase (BM_Tr_), while BM_An500_ and BM_An1,000_ designate 500 and 1,000 units/kg of extracted β-mannanase enzyme (BM_An_), respectively. Control group contains 0 units/kg of β-mannanase.

Two-way ANOVA indicated an insignificant effect (*P* > 0.05) of enzyme source and interaction (enzyme dosage × enzyme sources) on all biochemical indices of common carp. However, all pairwise comparisons indicated an almost similar positive effect of both enzymes (BM_An_ and BM_Tr_) on metabolic enzymes (AST and ALT activity) and serum protein, cholesterol, and triglyceride levels.

### Gene expression

**Figure 1:**
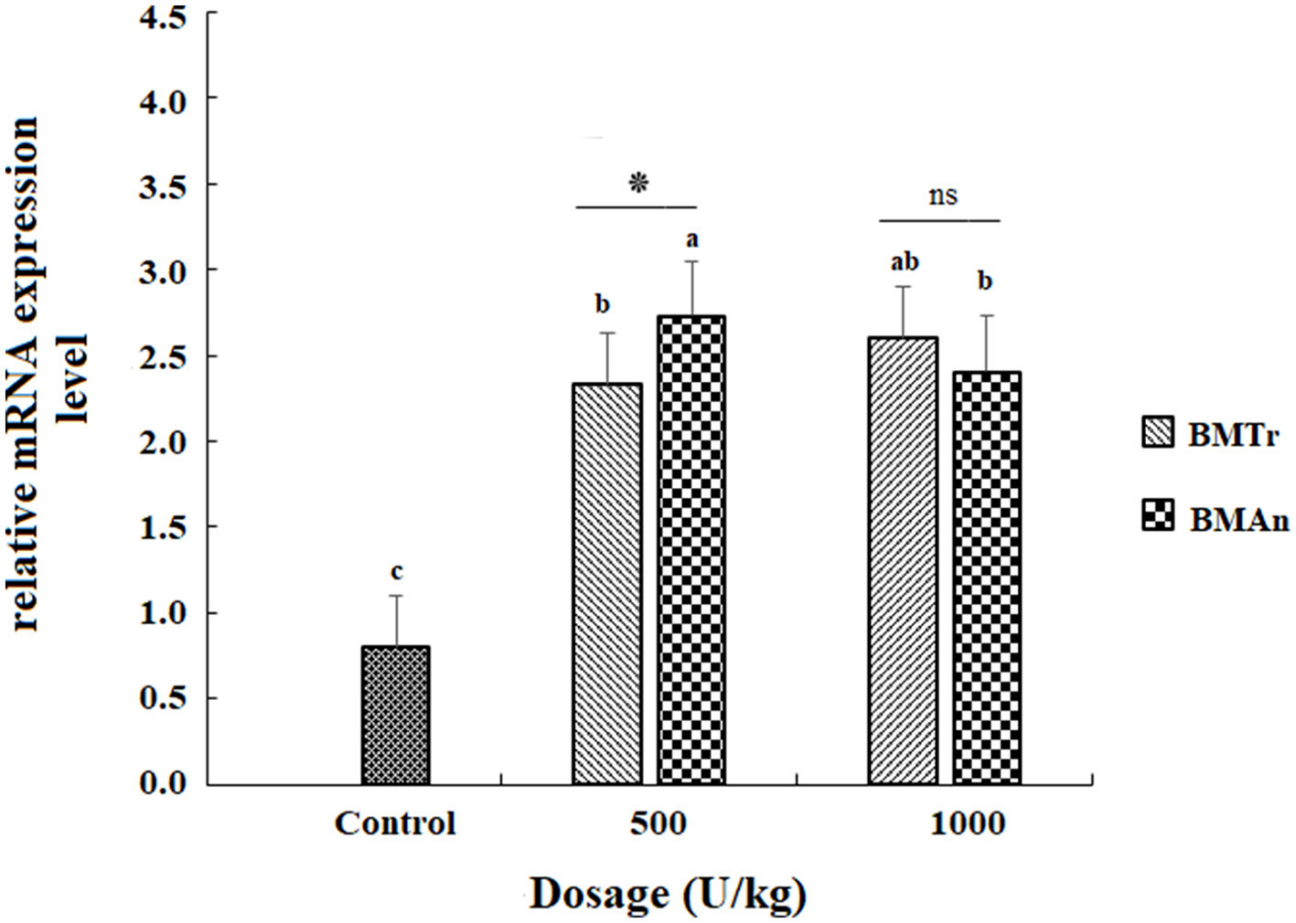
MyoD gene expression in the muscle of *C.carpio* fingerlings after 90 days of feeding β-mannanase supplemented diet. The bar shows the values as average ± SD, *n* = 9. ANOVA followed by LSD *post hoc* test represent comparisons between groups, while *t*-test compares the results of both enzymes (BM_Tr_ and BM_An_) at similar dosage level. Averages followed by different alphabets on bars are significantly different at *P* < 0.05. ns, non-significant, *P < 0.05. BM_Tr_, fermentation product of *Trichoderma reesei*. BM_An_, fermentation product of *Aspergillus niger*.

**Figure 2:**
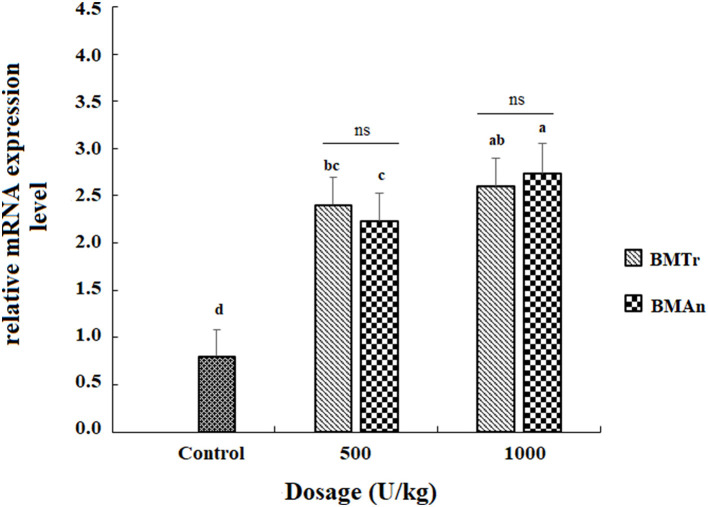
TNF-α gene expression in the liver of *C.carpio* fingerlings after 90 days feeding of β-mannanase supplemented diet. The bar shows the values as average ± SD, *n* = 9. ANOVA followed by LSD *post hoc* test represent comparisons between groups, while *t*-test compares the results of both enzymes (BM_Tr_ and BM_An_) at similar dosage level. Averages followed by different alphabets on bars are significantly different at *P* < 0.05. ns, non-significant, *P < 0.05. M_Tr_, fermentation product of *Trichoderma reesei*. BM_An_, fermentation product of *Aspergillus niger*.

**Figure 3:**
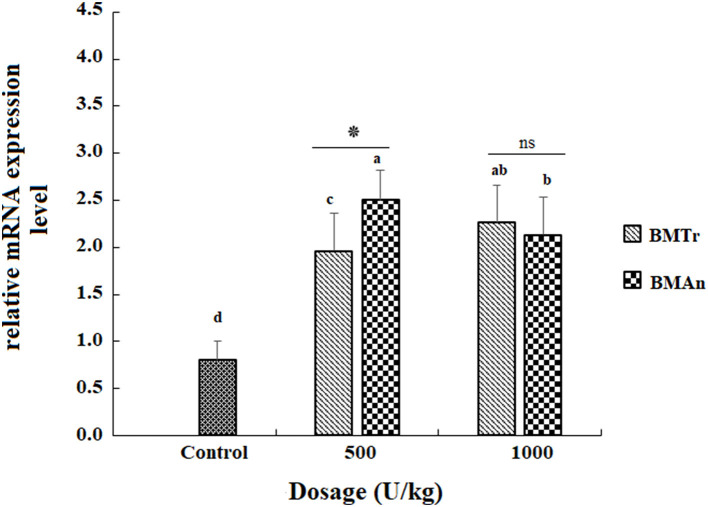
TNF-α gene expression in the intestine of *C. carpio* fingerlings after 90 days of feeding β-mannanase supplemented diet. The bar shows the values as average ± SD, *n* = 9. ANOVA followed by LSD *post hoc* test represent comparisons between groups, while *t*-test compares the results of both enzymes (BM_Tr_ and BM_An_) at similar dosage level. Averages followed by different alphabets on bars are significantly different at *P* < 0.05. ns, non-significant, *P < 0.05. BM_Tr_, fermentation product of Trichoderma reesei. BM_An_, fermentation product of *Aspergillus niger*.

**Figure 4:**
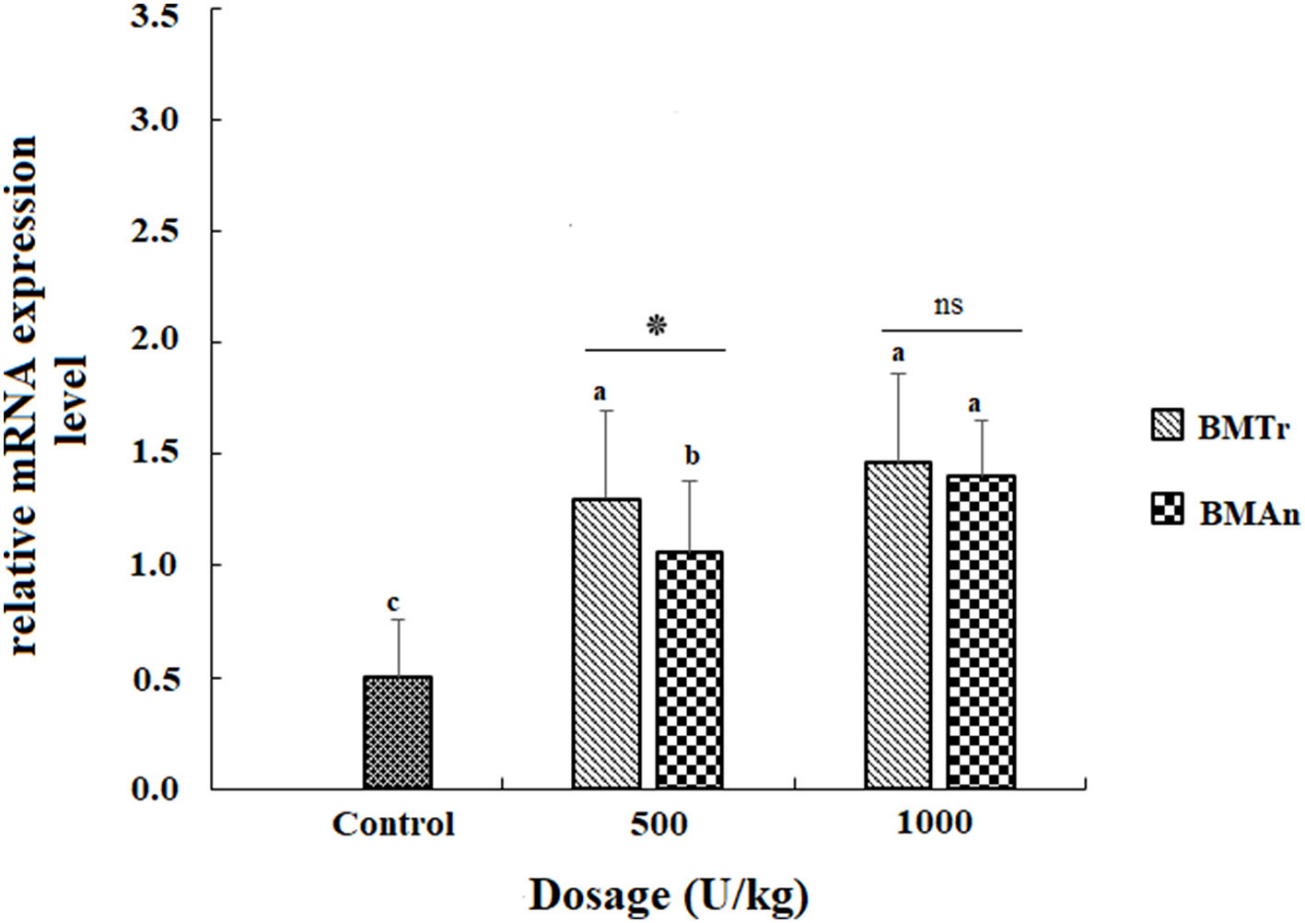
TNF-β gene expression in the muscle of *C. carpio* fingerlings after 90 days of feeding β-mannanase supplemented diet. The bar shows the values as average ± SD, *n* = 9. ANOVA followed by LSD *post hoc* test represent comparisons between groups, while *t*-test compares the results of both enzymes (BM_Tr_ and BM_An_) at similar dosage level. Averages followed by different alphabets on bars are significantly different at *P* < 0.05. ns, non-significant, *P < 0.05. BM_Tr_, fermentation product of *Trichoderma reesei*. BM_An_, fermentation product of *Aspergillus niger*.

The β-mannanase supplemented diet showed a significant positive effect on MyoD and TNF-α gene expression in different tissues of *C. carpio* fingerlings. The result indicated significantly higher expression of the MyoD gene in the muscle of *C. carpio* fed diets supplemented with the enzyme (Figure 1). Two-tailed tests indicated significantly higher MyoD expression in the muscle of BM_An500_ group (fed BM_An_ at the rate of 500 units kg-1 diet); however, at higher dosage level, that is, 1,000 units kg^−1^ diet, both enzymes showed a statistically similar effect. Similarly, TNF-α gene in the muscle, intestine, and liver of groups of fish fed β-mannanase supplemented diet also showed significantly higher expression (Figures 2–4) compared to a control group of fish. The pairwise comparison indicated that at higher dosage level (1,000 units kg^−1^ diet), both enzymes showed statistically similar effect, that is, higher expression of TNF-α gene in the muscle, liver, and intestine of *C. carpio;* however, at lower dosage level (500 units kg-1 diet), both enzymes (BM_An_ and BM_Tr_) showed a variable effect in different tissues.

## Discussion

The adverse effects of NSPs in plant protein-rich diets on the digestion of feed ingredients and the absorption of nutrients in different animals are well-documented (3, 39). Exogenous enzyme supplementation in the diet of monogastric animals has been reported to help alleviate these problems by reducing or eliminating the effects of NSPs (4, 40). Despite this, very few studies have been carried out to assess the effect of mannanase supplementation in fish diets. Here, we used the dietary β-mannanase enzyme to degrade mannan, a kind of NSP widely present in plant protein-rich diets, and we observed a significant positive effect of the exogenous enzyme on the growth, immunity, and muscle proximate composition of fish. We then compared the efficiency of β-mannanase isolated from two different microbial sources to determine which one performed better. We observed that the efficiency of β-mannanase locally extracted from *Aspergillus niger* AD-01 (BM_An_) was comparable to that of the commercial β-mannanase (BM_Tr_), a proprietary *Trichoderma reesei* fermentation product. Despite significant differences in the activity of BM_An_ and BM_Tr_ both enzymes demonstrated similar effects for various parameters studied. This could be due to a difference in pH profile, stability at low temperatures, or sensitivity to proteases. Similar results were obtained by other researchers when they investigated the comparative effect of two sources of phytase enzyme on chicken performance (41).

Although there is a substantial body of research demonstrating the beneficial effects of β-mannanase supplemented diets on different monogastric animals like pigs (15, 42) and poultry (17, 20, 43, 44), relatively few studies on fish (9, 21) have been reported to compare with our results. We found a significant and comparable increase in weight gain percentage and specific growth rate of *C. carpio* at both dosage levels (500 and 1,000 unit kg^−1^ diet) of β-mannanase compared to a control group. The upregulation of the MyoD gene in the muscle also indicated the positive effect of β-mannanase supplementation on the growth performance of carp. Our findings are in accord with Chen et al. (9), who observed similar beneficial effects on weight gain and specific growth rate of tilapia fed a 30% soybean meal (SBM) based diet supplemented with 4500 to 9000 U/kg of β-mannanase. Similarly, ali Zamini et al. (21) found that dietary β-mannanase (80,000 U/kg in combination with Natuzyme^®^) increased body weight gain and improved feed efficiency in *Salmo trutta caspius*. Our findings are also in agreement with many previous studies reported on other monogastric animals, such as broilers (18, 45), growing pigs (15, 46), and turkeys (16, 47). Some researchers, on the contrary, discovered no effect of a corn-SBM-based β-mannanase supplemented diet on broiler chicken's body weight gain and feed conversion (48). Similarly, Yigit et al. (49) also did not find a significant increase in the specific growth rate of trout in a 12-week trial in response to an SBM-based diet supplemented with β-mannanase at the dosage levels of 1 g/kg and 2 g/kg, alone or in combination with other carbohydrases. The discrepancy in the results may be attributed to a variety of factors, including temperature, age, species, mannan content in feed ingredients, and dosage levels of β-mannanase. It is well-documented that the efficiency of a dietary enzyme decreases as the water temperature decreases and increases with an increase in temperature toward an optimum range (50, 51).

In addition to the growth rate, we also observed a positive effect of β-mannanase on the FCR of *C. carpio*. Our findings support previous research that found enhanced FCR in tilapia (9) and broilers (19, 45, 52) in response to dietary β-mannanase. The improved growth performance and FCR observed here and reported by others may be due to an improvement in energy metabolism with the digestion of mannan or via degradation of mannan in feed to mannan oligosaccharides (MOS) and the utilization of these oligosaccharides as prebiotics by gut microbiota (9, 53, 54). Our results also indicated the significant positive effect of β-mannanase supplementation on the energy utilization and apparent digestibility of nutrients. It seems that β-mannanase degrades the indigestible fractions of a plant protein-rich diet and makes available the additional digestible energy. This results in a protein-sparing effect, leading to improved growth performance. The degradation of encapsulating indigestible fractions might also have increased the exposure of nutrient fractions to digestive enzymes (55, 56), thus resulting in improved digestibility of proteins and fats (Table 3) as observed in this study.

In this study, the observed significant increase in protein and fat content of the muscle of β-mannanase supplemented groups may indicate the beneficial effect of the dietary enzyme on the digestibility, absorption, and availability of nutrients. The higher moisture content and lower content of lipid and protein in the control group of fish may be due to the binding of mannan to water in the gut and the formation of viscous fluid, which prevents the contact of digestive enzymes with the substrate, thus reducing the digestibility and availability of nutrients (3, 4) for synthesis and deposition in the muscles. No available study shows the effect of β-mannanase on muscle composition. However, Hossain et al. (57) reported a reduction in whole-body lipid and gross energy contents and an increase in whole-body moisture of tilapia with an increase in sesbania endosperm (having high contents of storage mannan and other NSP) in feed. A few other investigators did not observe the effect of β-mannanase supplementation on carcass composition and whole-body composition of rainbow trout (49, 58), which may be due to low temperatures. It is well-established that enzyme activity is enhanced at higher water temperatures rather than at low water temperatures (50).

In this study, significantly increased levels of AST and ALT and decreased levels of CHO and TG in control group were observed, while an opposing trend in the β-mannanase supplemented groups was observed. This showed the negative effect of a plant protein-rich diet and the ameliorating effect of exogenous enzyme supplementation. Like our results, Chen et al. (9) also reported a lower level of serum AST and ALT in tilapia fed a β-mannanase supplemented diet. They, however, did not observe any significant effect on blood cholesterol, glucose, or HDL content. Studies report reduced lipid absorption and hypocholesterolemic effects of an NSP-containing plant protein-rich diet and the variable response of β-mannanase supplementation in different monogastric animals. Broilers fed a hemicell (β-mannanase) supplemented corn-SBM-based diet (59) and laying hens fed β-mannanase supplemented plant protein-rich diet (43) did not show any significant effect on CHO, TG, and very-low-density lipoprotein (VLDL). However, decreased serum LDL cholesterol level in response to β-mannanase in broilers was reported by Cho and Kim (44). The ratio of β-mannanase supplementation to the quantity of mannan in diet might be the cause of inconsistency in these findings (21). In this study, a dose-dependent increase in serum CHO and TG levels in the experimental groups may indicate enhanced fat absorption in the body. While hypocholesterolemia in the control group may be due to entrapment of bile salts by indigestible NSPs, this increased bile acid excretion may create an environment in which the body's cholesterol is being drawn out. In this state, cholesterol metabolism in the liver adjusts to provide cholesterol for enhanced bile acid synthesis.

Hematological indices are the key tools in assessing the changes in the physiology and health of fish. In this study, the improved blood indices like HB, HCT, RBCs, and MCHC in response to dietary β-mannanase may also indicate the better health status of fish. There is no comparable study on the effect of β-mannanase on the hematology of fish except for ali Zamini et al. (21) who observed no significant effect on hematological indices of Caspian salmon. However, Andrews et al. (60) reported the modulation of hematological indices in response to dietary MOS. The variation in the results can be attributed to the dosage level of β-mannanase, water temperature, or fish species.

The nutritional value of a diet depends on the digestive ability of the fish, which in turn is affected by its digestive enzyme activities. In this study, we observed a significant increase in the digestive enzyme (cellulase, protease, and amylase) activities of fish supplied with mannanase enzyme, as compared to a control group. Several studies have shown that supplementation of feed with exogenous enzymes can increase the production of endogenous enzymes (61–63). Although up till now, no study has reported the direct inhibitory effect of NSPs, including mannan, on intestinal enzyme activities, it has been reported that NSPs may restrict the access of digestive enzymes to substrates (3). Thus, the increased activity of digestive enzymes observed here and reported in other studies could be due to the absence of the inhibitory effects of NSPs on digestive enzymes by hydrolysis of NSPs or may also be due to the release of enzymes from gut microbiota that flourish in the presence of MOS (mannooligosaccharides, an end product of mannan degradation) (7).

In this study, we also observed a positive effect of β-mannanase supplementation on the immunity of fish, that is, a significant increase in IgM level, lysozyme activity, respiratory burst activity, and phagocytic activity of fish. Other researchers have documented similar immune response modulation in tilapia (9) and broilers (20, 64), suggesting that the hydrolytic product of mannan, that is, MOS, is indirectly stimulating the immune response. Generally, MOS act as prebiotics, block the colonization of pathogens in the GI tract by binding with mannose-specific type-I fimbriae, promote the growth of probiotics like *Bifidobacterium* and *Lactobacillus* species, and trigger the immune system by activation of pattern recognition receptors and protein (65). Many studies have found that MOS administration improves immune-related indices in a variety of fish and shellfish species, including grass carp (*Ctenopharyngodon idella*) (66), red sea beam (*Pagrus major*) (67), Japanese flounder (*Paralichthys olivaceus*) (68), crucian carp (*Carassius auratus gibelio*) (69), European sea bass (*Dicentrarchus labrax)* (70–72), crustaceans (*Litopenaeus vannamei*) (73, 74), sea cucumber (*Apostichopus japonicus*) (75), and other vertebrates, for example, broilers (76) and pigs (77).

Here, we also observed upregulation of TNF-α gene in the intestine and muscle of all the groups of *C. carpio* fed mannanase supplemented diet. TNF-α was chosen because it is a pleiotropic cytokine produced predominantly by macrophages and plays an important role in the innate immune response, homeostasis regulation, hematopoiesis, and lymphocyte survival (78). TNF-α activates macrophages, increasing the rate of phagocytosis, respiratory burst activity, and nitric oxide production (79). There is no comparable study that reports TNF-α gene regulation in fish following β-mannanase supplementation. Many researchers, however, found a significant increase in the mRNA level of this gene in Nile tilapia (80, 81) and common carp (35) fed probiotics (*Spirulina platensis, Bacillus amyloliquefaciens*, and *Lactobacillus rhamnosus*) and prebiotics (galacto/fructooligosaccharide and inulin) supplemented diets, respectively. The observed higher expression of TNF-α gene in the present study may indicate the resistance of fish to infection (82).

## Conclusion

In conclusion, β-mannanase supplementation irrespective of microbial origin showed a beneficial effect on the apparent digestibility coefficient of nutrients, the growth, body composition, intestinal enzyme activity, hemato-immunological indices, metabolic enzymes, and expression of growth and immune-related genes of *C. carpio*. Based on the findings, 500 U/kg β-mannanase supplementation may be recommended for improved carp production on low-cost feed, that is, plant protein-rich diet where fish meal, an expensive source of protein, is replaced by the cheapest and easily available plant source of protein.

## Data availability statement

The original contributions presented in the study are included in the article/supplementary material, further inquiries can be directed to the corresponding author/s.

## Ethics statement

The animal study was reviewed and approved by Quaid-i-Azam University's animal welfare committee.

## Author contributions

AD conceived and designed the study with the help of WS. AD executed the experiments. Both the authors critically revised the manuscript for important intellectual contents and approved the final version. Both authors contributed to the article and approved the submitted version.

## Conflict of interest

The authors declare that the research was conducted in the absence of any commercial or financial relationships that could be construed as a potential conflict of interest.

## Publisher's note

All claims expressed in this article are solely those of the authors and do not necessarily represent those of their affiliated organizations, or those of the publisher, the editors and the reviewers. Any product that may be evaluated in this article, or claim that may be made by its manufacturer, is not guaranteed or endorsed by the publisher.
